# 20680 Characterization of Clinical and Immunological Laboratory Features in Multiple Sclerosis Patients with COVID-19

**DOI:** 10.1017/cts.2021.767

**Published:** 2021-03-30

**Authors:** Ahya S Ali, Yang Mao-Draayer

**Affiliations:** Autoimmunity Center of Excellence, University of Michigan Medical School

## Abstract

ABSTRACT IMPACT: Better understanding of the factors impacting disease severity and immunological response of MS patients on disease modifying therapy will enable better recommendations for vaccination options and risk mitigation strategies OBJECTIVES/GOALS: The Coronavirus Disease 2019 (COVID-19) and global health crisis has raised health concerns for patients with multiple sclerosis (MS). We aim to study the clinical characteristics, immunological laboratory data, and immunoglobulin response in patients with MS and COVID-19, to identify factors impacting disease severity and immune response. METHODS/STUDY POPULATION: Database search was done using DataDirect to search for MS patients who had tested positive for COVID-19 at the University of Michigan hospital. Patients with a positive nasopharyngeal swab polymerase chain reaction (PCR) for COVID-19 between March 1 and September 2020 were included. The primary outcome was the immunological laboratory data and immunoglobulin levels and the secondary outcome was their disease severity. We collected demographics, neurological history, MS treatment, Expanded Disability Scale Score (EDSS), comorbidities, and COVID-19 characteristics. A 7-point ordinal scale previously used to assess disease severity was used. Univariate and multivariate analyses will be performed to assess relationships between the collected variables. RESULTS/ANTICIPATED RESULTS: A total of 17 patients, mean age 53 (SD 11.6) years, mean disease duration, 6.2(SD 4.1) years were analyzed. 41% of patients had relapsing remitting multiple sclerosis, 17% had primary progressive MS. (88%) patients were on Disease Modifying Therapy (DMT) at the time of COVID-19 diagnosis. 2 patients died from COVID-19 complications. There was a higher proportion of patients with higher disease severity receiving Ocrelizumab. Only one patient showed positive IgG to SARS-CoV-2 after the resolution of infection. CBC with differential was obtained and a longitudinal follow-up of labs will be done. Regression analysis will be done to check the association between the use of DMT, immunological response, and COVID disease severity in them. The impact of COVID-19 on MS relapse, EDSS, and MRI activities will also be studied. DISCUSSION/SIGNIFICANCE OF FINDINGS: Recommendations to continue current DMT have been made, however, the immune response has not been correlated with the individual’s risk profile. Certain therapies may interfere with mounting a protective immune response of COVID-19 and this knowledge is crucial when advising patients regarding the choice of vaccine and risk mitigation strategies.

